# Deep Reinforcement Learning-Based Automated Treatment Planning for a Rotating Gamma System

**DOI:** 10.7759/cureus.109111

**Published:** 2026-05-18

**Authors:** Jinsheng Li, Peng Guo, Xiangyu Guo

**Affiliations:** 1 Radiation Oncology, University of Miami Miller School of Medicine, Miami, USA; 2 Treatment Planning, OUR United Corporation, Xi’an, CHN; 3 Medicine, Xi’an International Medical Center, Xi’an, CHN

**Keywords:** automated planning, deep reinforcement learning, gamma knife stereotactic radiosurgery, rotational gamma system, srs

## Abstract

Introduction

Traditional treatment planning for the rotating gamma system (RGS) is a complex and time-consuming process that relies heavily on planner experience. Manual selection of shot size, location, and weight requires extensive trial and error to achieve adequate target coverage (Cov) and dose conformity.

Methods

We developed an automated treatment planning algorithm for RGS using deep reinforcement learning (DRL). Shot placement was modeled as a sequential decision-making process based on target geometry and cumulative dose distribution. A proximal policy optimization (PPO) algorithm was implemented, using target dose Cov and conformal index as reward-related parameters. Thirty clinical brain tumor cases with target volumes ranging from 0.5 to 45 cc were used for evaluation. An additional 15 cases that were not included in the training were used for independent validation.

Results

The DRL-based algorithm generated clinically acceptable treatment plans across a wide range of tumor sizes. Average dose Cov and selectivity were 92% and 0.79 for tumors <1 cc, 92% and 0.82 for tumors 1-2 cc, 93% and 0.87 for tumors 2-20 cc, and 93% and 0.81 for tumors >20 cc. In the independent test cohort, average Cov and selectivity were 96% and 0.81, respectively. Comparisons with clinical treatment plans created by an experienced planner demonstrated no statistically significant differences in Cov, selectivity, and conformity index (CI, Radiation Therapy Oncology Group (RTOG) criteria), although further improvement in conformity remains desirable. Plan generation time for a single target was less than 10 minutes.

Conclusion

The proposed DRL-based automated planning algorithm demonstrates the feasibility of generating RGS treatment plans. The approach achieves plan quality comparable to planner-driven methods while significantly reducing planning time and reliance on user expertise. These results highlight its strong potential to improve efficiency and consistency in stereotactic radiosurgery (SRS) planning.

## Introduction

Stereotactic radiosurgery (SRS) and stereotactic radiotherapy (SRT) using stationary and rotating gamma systems (RGS), such as the Gamma Knife (GK) [1] and RGS [2], are well-established treatment modalities for intracranial tumors and selected extracranial lesions [3,4]. These systems deliver highly conformal radiation doses through multiple radioactive sources, enabling effective tumor control while sparing surrounding normal tissues.

Despite their clinical effectiveness, treatment plans for gamma-based systems are mainly generated based on manual forward planning, even though most treatment planning systems have an inverse planning technique implemented. Medical physicists must sequentially place multiple radiation shots, selecting their size, spatial location, and relative weight based on three-dimensional (3D) imaging of the target during the forward planning process. Each decision influences the resulting dose distribution, necessitating repeated adjustments to optimize target coverage (Cov) and dose conformity. This planner-driven workflow is time-consuming, experience-dependent, and subject to inter-planner variability.

The planning process for gamma systems is inherently sequential, with each action affecting subsequent decisions and long-term outcomes. These characteristics closely align with reinforcement learning (RL) [5], a class of artificial intelligence (AI) methods in which an agent learns optimal actions through trial-and-error interactions with an environment guided by a reward function. Similar to strategic board games such as Go [6], gamma system planning is deterministic, fully observable, and involves delayed rewards, making it well-suited for RL-based automation.

Recent advances in AI, particularly deep learning and deep reinforcement learning (DRL), have driven rapid progress in radiation oncology, including applications in image segmentation, treatment planning, delivery optimization, and outcome prediction [6-9]. Compared with traditional rule-based or optimization-driven planning approaches, DRL can learn complex planning strategies directly from data without extensive manual parameter tuning [10-14].

The objective of this study was to evaluate the feasibility of a proximal policy optimization (PPO)-based DRL framework for generating clinically acceptable RGS treatment plans with acceptable Cov and conformity in a time frame comparable to planner-driven manual planning. We developed a DRL-based automated planning algorithm using PPO. By modeling shot placement as a sequential decision-making process guided by target geometry and dose distribution, the algorithm aimed to generate clinically acceptable plans with minimal human intervention. Feasibility was assessed based on plan quality and planning efficiency using a diverse set of clinical brain tumor cases.

## Materials and methods

Treatment planning formulation

**Figure 1:**
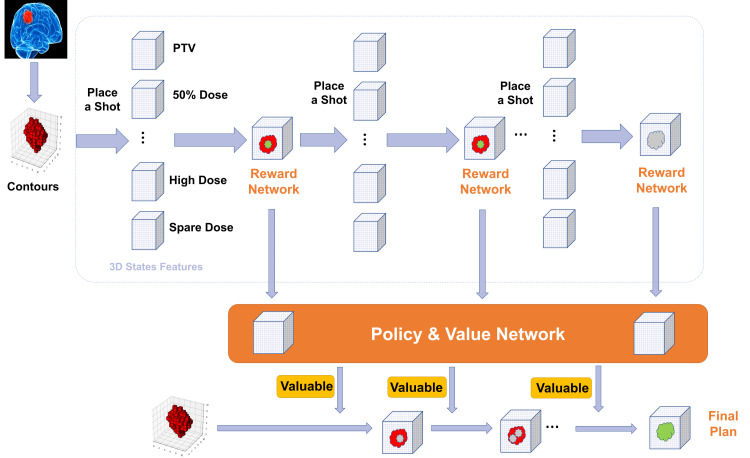
Workflow of DRL-based auto planning. Place shots sequentially and calculate their values based on the 3D feature and shot placement policy. The shot is accepted if it is most valuable based on the current situation. This image is an original, author-created schematic using PowerPoint (Microsoft® Corp., Redmond, WA, USA) and was not generated using AI. DRL: Deep Reinforcement Learning

The workflow of the DRL-based automated planning algorithm is illustrated in Figure 1. The inputs include the target and organ-at-risk (OAR) volumes, along with the patient’s 3D imaging data. The image and volume data were downsampled to a spatial resolution of 1 mm in all three dimensions. Automated treatment planning was formulated as a sequential decision-making process. At each decision step, the agent selects a radiation shot characterized by its collimator size, 3D location, and weight. The probability of selecting each shot size at a given state is determined by the agent based on the current state features.

The state space incorporates both target geometry and the accumulated dose distribution. Specifically, the state features include the target mask, dose mask, and feasibility mask, which represent the target volume, the prescription dose volume, and the difference between the two in 3D space, respectively. The dose calculation was performed with a TMR (tissue maximum ratio)-based dose calculation algorithm. Measured dose profiles at different depths were utilized to calculate the off-axis dose. 

The reward function integrates target dose Cov and the selectivity index (SI) to promote high target conformity while minimizing dose spillage to surrounding tissues. Cov is defined as the percentage of the target volume receiving at least the prescription dose. The SI is defined as the ratio of the target volume covered by the prescription (or higher) dose to the total irradiated volume receiving the prescription (or higher) dose. The reward function was defined as a weighted combination of improvements in target Cov and selectivity, together with a penalty term representing the percentage of prescription-dose volume outside the target. The weighting factors were empirically tuned through iterative experimentation.

After each new shot is added, increases in Cov and SI are assigned as positive rewards, while the proportion of the prescription dose volume outside the target is penalized as a negative reward. The planning episode terminates once predefined criteria for target Cov and conformity are satisfied.

DRL framework

**Figure 2:**
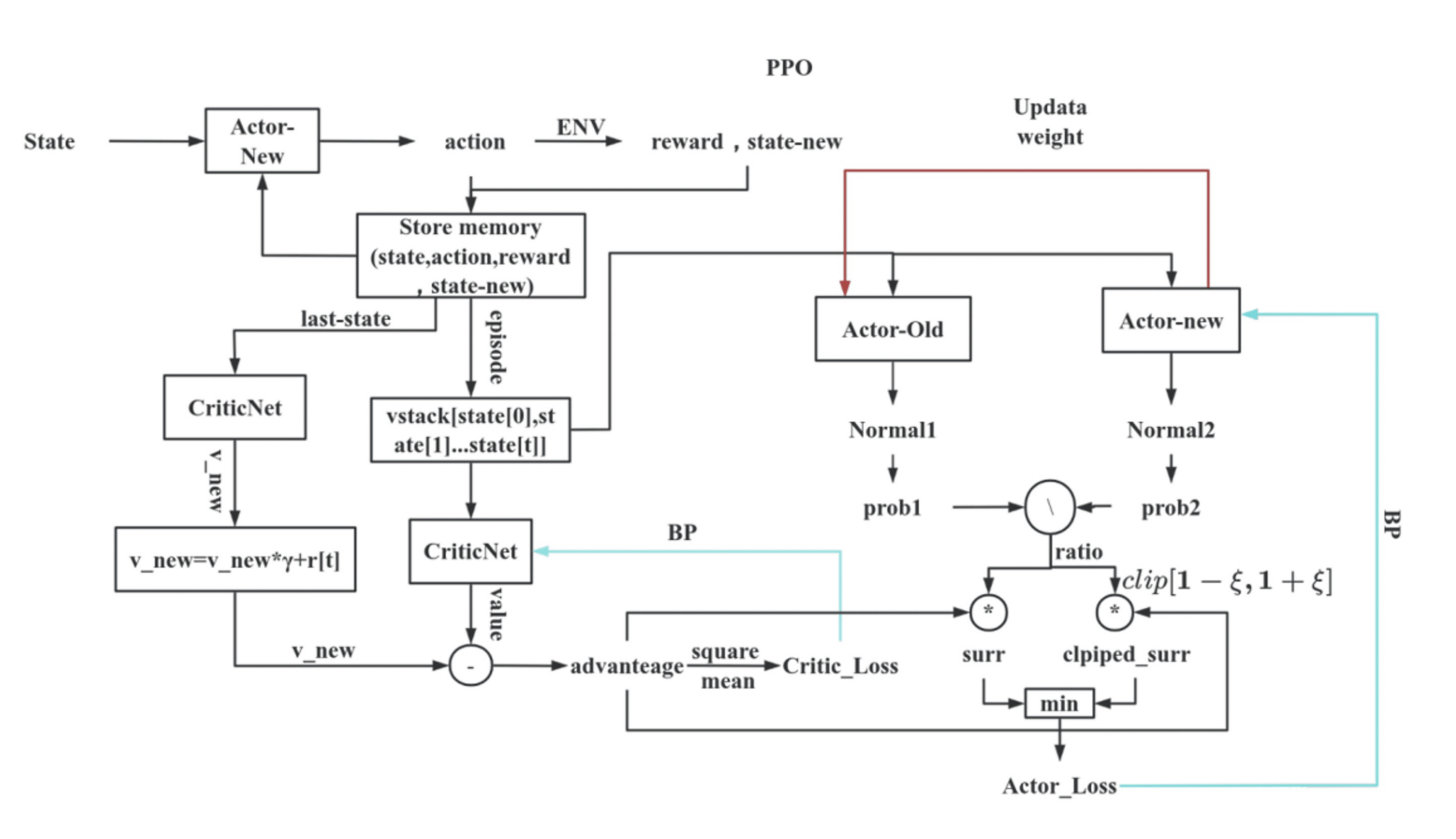
Actor-Critic architectural framework based on the PPO policy gradient algorithm, comprising an ActorNet, a CriticNet, a PPOMemory module, and an Agent. PPO: Proximal Policy Optimization

**Figure 3:**
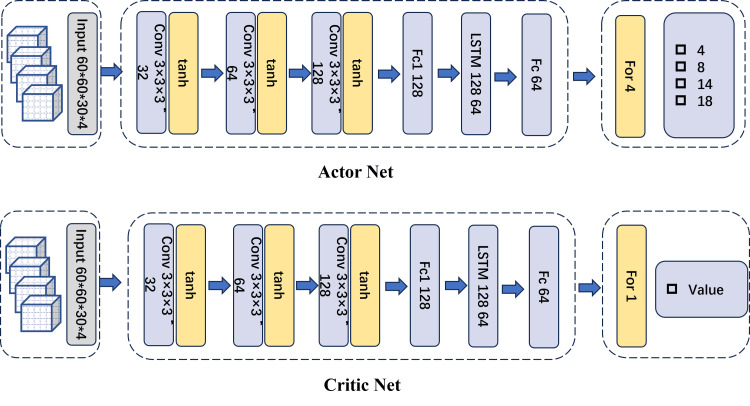
Actor Net and Critic Net. This image is an original, author-created schematic using PowerPoint (Microsoft® Corp., Redmond, WA, USA) and was not generated using AI.

A PPO algorithm [15] was implemented to train the planning agent. PPO is a policy-gradient method that enhances training stability by constraining the magnitude of policy updates using a clipped objective function. An actor-critic architectural framework based on the PPO algorithm was adopted, comprising an ActorNet, a CriticNet, a PPOMemory module, and an Agent, as illustrated in Figures 2-3.

During training, the Actor network (“Actor-New”) interacts with the environment to sample actions and collect experience in the form of state-action-reward-next-state tuples. The Critic network evaluates each state by estimating its value function and computing the advantage function, defined as the difference between the observed return and the expected value.

Policy updates are performed iteratively by comparing the action probabilities of the updated (Actor-New) and previous (Actor-Old) networks. A clipping mechanism is applied to constrain the update magnitude, and the minimum surrogate objective is used to update the Actor network. Concurrently, the Critic network is optimized by minimizing the mean squared error (MSE) between predicted and target values using the Adam optimizer provided by Python. Through this iterative process, the agent progressively improves its policy while maintaining stable and efficient learning.

The ActorNet outputs a probability distribution over all possible actions given the current state. A SoftMax layer is applied to normalize these probabilities and select the action with the highest likelihood. To reduce the complexity of the action search, the convolutional output is multiplied by an executable action space mask prior to the SoftMax operation, thereby restricting the probability calculation to valid actions only.

The Actor network consists of four convolutional layers, followed by a SoftMax layer that outputs the probability distribution for selecting one of four collimator sizes under the current state. The input to the network comprises four 3D binary mask matrices (values of 0 or 1): State0, State1, State2, and State3. Specifically, State0 = PTV_mask - dose_mask (i.e., the planning target volume (PTV) minus the 50% isodose distribution from the previous step), State1 = dose_mask (the 50% isodose distribution from the previous step), State2 = OAR_mask (organs at risk), and State3 = overdose_mask (regions outside the target receiving excessive dose). The difference between PTV_mask and dose_mask (State0), also referred to as the feasibility mask, is used to constrain the action space by prohibiting shot placement outside feasible regions, thereby avoiding non-meaningful actions.

The Critic network processes the same input through four convolutional layers and outputs the estimated value of the current state via a linear layer. It evaluates the action selected by the ActorNet at time step t by estimating the state-value function. The PPO algorithm additionally records the policy probability distribution π for each episode.

The PPOMemory module stores the state, action probabilities, state values, selected actions, rewards, and terminal flags (“done”) for each batch. The overall loss function is defined as a weighted sum of the actor loss and critic loss.

Through repeated interactions with the planning environment, the neural network learns to map observed states to optimal actions. No manual rules or handcrafted heuristics are imposed, allowing the agent to autonomously determine shot combinations, locations, and weights.

The network employed 3D convolutional layers with a kernel size of 3, stride of 1, and padding of 1 throughout. The PPO algorithm was trained using five epochs per update with a batch size of 8. Training parameters were set as follows: learning rate = 0.003, reward discount factor (γ) = 0.99, and clipping parameter (ε) = 0.2. The number of training iterations for each case was set to 1000, with each training episode requiring approximately three to five minutes.

Patient data

Images and contours of 30 clinical brain tumor patients were randomly selected retrospectively for training and evaluation. All patients have been treated in three GK centers. No selection criteria were set. The target volume ranged from 0.5 to 45 cc, and these cases included metastases, pituitary tumors, meningiomas, and multi-target metastases. Targets and organs at risk were delineated on MRI or CT images according to standard clinical practice. All cases were planned with shots using full-arc irradiation for treatment on an RGS, even though partial arc irradiation is available for the RGS under study.

After training, 15 additional brain tumor cases from another GK Center that were not included in the training dataset were used for independent testing. These cases included meningiomas, pituitary adenomas, and metastases with target volumes ranging from 0.3 to 6.7 cc.

Data analysis and evaluation metrics

Plan quality was assessed using dose Cov and conformity index (CI). The CI defined by the Radiation Therapy Oncology Group (RTOG) [4] is the ratio of the prescription isodose volume to the target volume and equals Cov divided by selectivity based on their definitions. The CI defined by Paddick [16] equals Cov multiplied by selectivity. Planning efficiency was evaluated based on total plan generation time. The automated output included shot size combinations, shot positions, shot weights, 3D dose distributions, and dose-volume histograms (DVHs). The results for the 15 independent test cases were also compared with the results of planner-driven forward planning that was performed by an experienced medical physicist.

## Results

Overall plan quality

The DRL-based algorithm generated clinically acceptable treatment plans across all tumor size categories for the 30 patients that were used for training and evaluation, demonstrating consistently high dose Cov and conformality as shown in Table 1. The plans were always normalized to the 50% isodose line. It can be adjusted based on clinical requirements for Cov and conformality for any individual case.

**Table 1:** Summary of automated RGS treatment planning results stratified by target volume. The average values of plan quality, including coverage, selectivity, and CI, for different target volume categories are shown. RGS: Rotating Gamma System; CI: Conformity Index; RTOG: Radiation Therapy Oncology Group

Target Volume Category	Average Dose Coverage (%)	Average Selectivity	Average CI (RTOG)	Average CI (Paddick)
< 1 cc	92	0.79	1.16	0.73
1-2 cc	92	0.82	1.12	0.75
2-20 cc	93	0.87	1.07	0.81
> 20 cc	93	0.81	1.15	0.75

Independent validation

In the independent test cohort, the automatically generated plans achieved an average dose Cov of 96% and an average selectivity of 0.81 across the 15 test cases, as shown in Table 2. The mean CIs were 1.18 and 0.78 according to the RTOG and Paddick definitions, respectively. Nine cases met or exceeded the clinical thresholds of Cov ≥ 95% and selectivity ≥ 0.8.

**Table 2:** Test results of independent validation for DRL-based auto planning. The target sizes and comparison with manual planning on plan qualities, indicated by coverage, SI, and CI, are shown. DRL: Deep Reinforcement Learning; SI: Selectivity Index; CI: Conformity Index; RTOG: Radiation Therapy Oncology Group

ID	Volume (cc)	Coverage (%)		SI		CI (RTOG)		CI (Paddick)	
		Manual	Auto	Manual	Auto	Manual	Auto	Manual	Auto
1026002	6.7	96	96	0.9	0.76	1.07	1.26	0.86	0.73
1026003	1.5	96	98	0.86	0.8	1.12	1.23	0.83	0.78
1026004	0.3	96	96	0.8	0.79	1.20	1.22	0.77	0.76
1026006	0.9	96	98	0.85	0.75	1.13	1.31	0.82	0.74
1026007	2.9	96	98	0.86	0.8	1.12	1.23	0.83	0.78
1026009	3.7	95	95	0.78	0.84	1.22	1.13	0.74	0.80
1026010	1.5	99	96	0.85	0.77	1.16	1.25	0.84	0.74
1026011	3.7	98	94	0.85	0.87	1.15	1.08	0.83	0.82
1026012	2.2	95	97	0.84	0.79	1.13	1.23	0.80	0.77
1026021	3.4	95	94	0.83	0.88	1.14	1.07	0.79	0.83
1026031	0.4	96	97	0.83	0.76	1.16	1.28	0.80	0.74
1026032	1.7	99	95	0.81	0.88	1.22	1.08	0.80	0.84
1026034	2.9	95	95	0.9	0.86	1.06	1.10	0.86	0.82
1026035	0.7	95	93	0.82	0.83	1.16	1.12	0.78	0.77
1026037	2.5	95	95	0.88	0.81	1.08	1.17	0.84	0.77
Average	2.33	96.1	95.7	0.844	0.813	1.141	1.181	0.811	0.778

Comparisons with manually generated clinical treatment plans created by an experienced clinical physicist were also shown in Table 2 and demonstrated no statistically significant differences in Cov, SI, and CI (RTOG), with p-values of 0.472, 0.071, and 0.144, respectively, although further improvement in conformity remains desirable since the p-value for CI (Paddick) is only 0.028. The average number of shots used in automated planning was 9.8, compared with 9.5 for planner-driven forward planning.

Representative clinical cases

**Figure 4:**
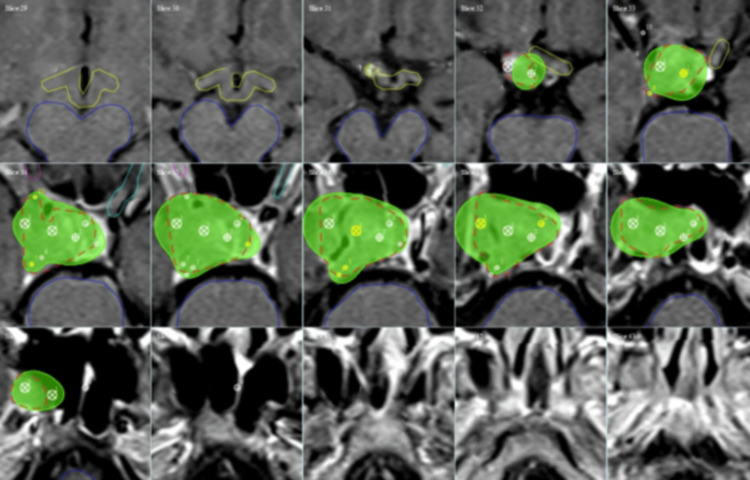
Automatically generated RGS treatment plan for a 6.7 cc target, achieving 96% dose coverage at the 50% isodose line and a selectivity index of 0.76. The dashed red lines indicate the target volume, and the prescription dose cloud is shown in green. RGS: Rotating Gamma System

**Figure 5:**
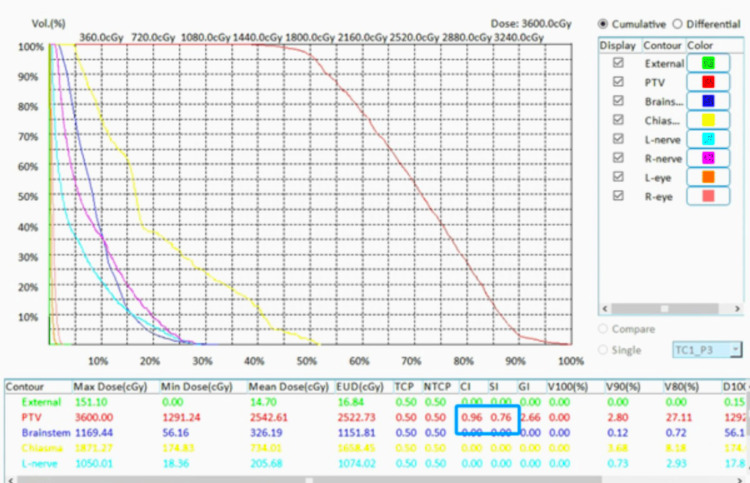
DVHs and plan quality indexes for the plan shown in Figure 4. DVH: Dose-Volume Histogram

**Figure 6:**
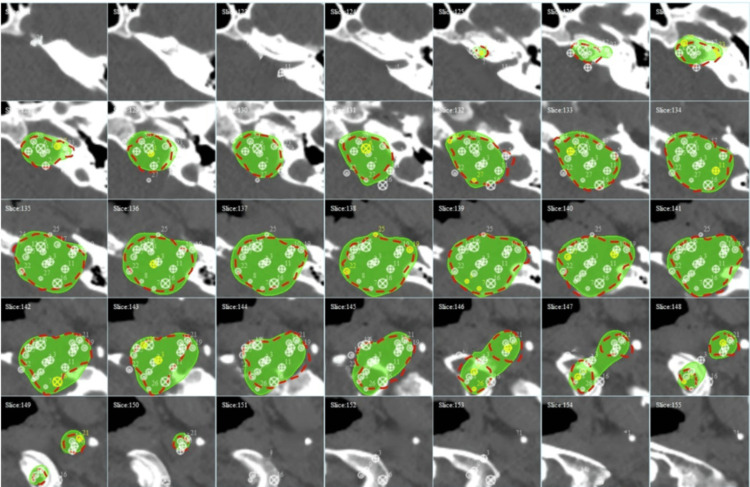
Automatically generated RGS treatment plan for a 10.1 cc target, achieving 96% dose coverage at the 50% isodose line and a selectivity index of 0.81. The dashed red lines indicate the target volume, and the prescription dose cloud is shown in green. RGS: Rotating Gamma System

**Figure 7:**
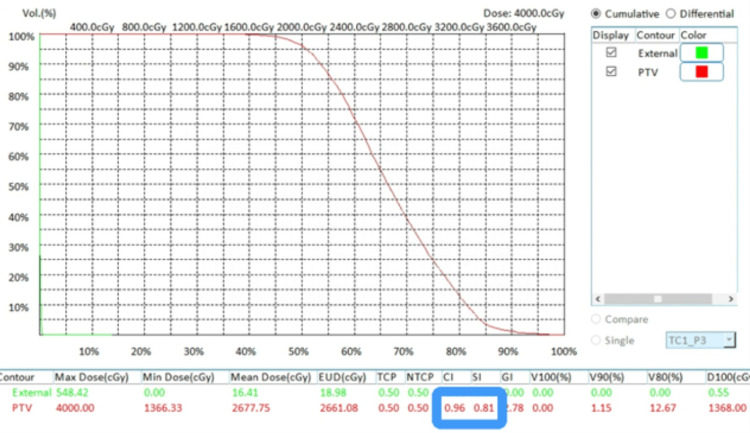
DVHs and plan quality indexes for the plan shown in Figure 6. DVH: Dose-Volume Histogram

The dose cloud Cov of one automatically generated RGS treatment plan for a large brain tumor is shown in Figure 4 to illustrate dose Cov and conformality. The corresponding DVHs and plan quality indices are shown in Figure 5. The dose cloud Cov of another automatically generated RGS treatment plan for a large brain tumor is shown in Figure 6 to illustrate dose Cov and conformality. The corresponding DVHs and plan quality indices are shown in Figure 7. The first case is a pituitary patient with a tumor size of 6.7 cc. The plan was generated with nine shots, achieving 96% dose Cov at the 50% isodose line and a SI of 0.76. The RTOG and Paddick CI values are 1.26 and 0.73, respectively. The second case is a metastasis with a tumor size of 10.1 cc. The autogenerated plan used 15 shots and achieved 96% dose Cov at the 50% isodose line and a SI of 0.81. The RTOG and Paddick CI values are 1.18 and 0.78, respectively. The dashed red lines indicate the target volumes, and the prescription dose cloud is shown in green.

Planning efficiency

All automated plans were generated in less than 10 minutes per target for most cases using a laptop workstation equipped with an Intel Core i7-14650HX processor (2.2 GHz base frequency), 16 GB RAM, and an NVIDIA GeForce RTX 4060 GPU, demonstrating substantial time savings compared with manual planning workflows. Comparison of planning time is shown in Table 3 for the 15 independent test cases.

**Table 3:** Comparison on planning time between automated planning and planner driven manual planning for the 15 test cases.

ID	Cancer Type	Manual (minutes)	Auto (minutes)
1026002	Pituitary tumor	35.72	7.6
1026003	Pituitary tumor	43.62	10.0
1026004	Pituitary tumor	45	3.5
1026006	Pituitary tumor	23.2	5.9
1026007	Meningioma	26	5.5
1026009	Meningioma	30.1	5.3
1026010	Meningioma	50.93	9.6
1026011	Meningioma	46.78	9.1
1026012	Meningioma	47.25	6.8
1026021	Meningioma	51.62	3.8
1026031	Metastases	23.83	4.2
1026032	Metastases	19.82	7.1
1026034	Metastases	29.7	6.3
1026035	Metastases	34.5	3.8
1026037	Metastases	24.42	6.0

## Discussion

Applications of AI in radiotherapy can generally be categorized into three domains: (1) pre-treatment tumor characterization and segmentation, (2) radiotherapy/radiosurgery treatment planning, and (3) post-treatment response prediction. Among these areas, applications in automatic treatment planning (ATP) are the focus of this study and have been discussed and reviewed by several investigators. Wang et al. [14] reviewed previous efforts on AI applications in ATP and explored future possibilities. Their work mainly focused on the general principles and algorithms underlying AI-based treatment planning. Most of the research directions they summarized involve auto-planning for linear accelerator-based radiotherapy, including spatial dose prediction methods [17-19] used for knowledge-based modeling and planning. In contrast, applications for GK-type radiosurgery planning remain relatively limited.

Liu et al. [20] reported a study on automatic inverse treatment planning for GK radiosurgery using DRL. In their work, a DRL-based priority-tuning policy network was developed to automatically adjust the priorities among planning objectives in order to improve the efficiency of GK inverse planning. The AI algorithm was primarily used to determine objective priorities during the optimization process, thereby improving the efficiency of inverse planning. Their method was tested on vestibular schwannoma cases and achieved planning scores comparable to those produced by expert manual planners, demonstrating its potential utility in routine clinical practice.

However, their approach mainly functions as an assistant to the inverse planning optimization process by adjusting objective priorities. To date, no published studies have reported an intelligent agent capable of performing shot placement in a manner similar to a human expert - that is, automatically selecting both the collimator size and shot locations within the tumor for all required shots and optimizing their weights to achieve an optimal treatment plan with good Cov and conformity.

This study demonstrates the feasibility and effectiveness of applying DRL to automatically select and place shots for RGS treatment planning. By formulating shot placement as a sequential decision-making problem, the proposed algorithm is able to generate plans comparable to operator-driven plans across a wide range of tumor sizes and pathologies.

Compared with conventional manual planning, the DRL-based approach significantly reduces the need for iterative trial-and-error processes and dependence on planner experience. Unlike traditional optimization-based automated planning methods, the proposed framework does not rely on handcrafted rules or extensive parameter tuning, allowing the model to adapt flexibly to different target geometries.

The comparable performance between automated and manually generated plans, particularly for small and irregularly shaped targets, highlights the clinical potential of this approach. The relatively short plan generation time further supports its applicability in routine clinical workflows.

In this study, only treatments with full-arc rotation were considered during both training and evaluation for RGS auto-planning. Optimization of beam angle sector selection may be incorporated in future work. Retrospective data from a rather limited number of centers may be subject to selection bias. Future validation on multi-institutional datasets is key to strengthening generalizability. In addition, the efficiency of the DRL-based planning process still requires further improvement for broader clinical implementation, which could be achieved through enhancements in both algorithm design and computational hardware. 

## Conclusions

A DRL-based automated treatment planning algorithm for the RGS was developed and validated. Although further improvement in dose conformity is warranted, the proposed method is capable of generating treatment plans comparable to those created by experienced planners while substantially reducing planning time and inter-planner variability. These findings demonstrate the feasibility of DRL-based automated planning and highlight its potential for future clinical implementation in SRS.
